# Avian ecosystem functions are influenced by small mammal ecosystem engineering

**DOI:** 10.1186/1756-0500-6-549

**Published:** 2013-12-20

**Authors:** Meredith Root-Bernstein, Andres Fierro, Juan Armesto, Luis A Ebensperger

**Affiliations:** 1 Department of Ecology, Facultad de Ciencias Biologicas, Pontificia Universidad Católica de Chile, Santiago, Chile; 2 Oxford University Centre for the Environment, School of Geography and the Environment, Oxford University, Oxford, UK; 3 Department of Ecological Sciences, Facultad de Ciencias, Universidad de Chile, Santiago, Chile; 4 Institute of Ecology and Biodiversity, Box 653, Santiago, Chile

**Keywords:** Avian, Foraging, Functional diversity, Mobile link species, *Octodon degus*, Runways

## Abstract

**Background:**

Birds are important mobile link species that contribute to landscape-scale patterns by means of pollination, seed dispersal, and predation. Birds are often associated with habitats modified by small mammal ecosystem engineers. We investigated whether birds prefer to forage on degu (*Octodon degus*) runways by comparing their foraging effort across sites with a range of runway densities, including sites without runways. We measured granivory by granivorous and omnivorous birds at Rinconada de Maipú, central Chile. As a measure of potential bird foraging on insects, we sampled invertebrate prey richness and abundance across the same sites. We then quantified an index of plot-scale functional diversity due to avian foraging at the patch scale.

**Results:**

We recorded that birds found food sources sooner and ate more at sites with higher densities of degu runways, cururo mounds, trees, and fewer shrubs. These sites also had higher invertebrate prey richness but lower invertebrate prey abundance. This implies that omnivorous birds, and possibly insectivorous birds, forage for invertebrates in the same plots with high degu runway densities where granivory takes place. In an exploratory analysis we also found that plot-scale functional diversity for four avian ecosystem functions were moderately to weakly correllated to expected ecosystem function outcomes at the plot scale.

**Conclusions:**

Degu ecosystem engineering affects the behavior of avian mobile link species and is thus correlated with ecosystem functioning at relatively small spatial scales.

## Background

The relatively high mobility of birds at a landscape scale gives them an important role in linking ecological processes across space [1,2]. Through foraging activity, for example, birds can act as pollinators, seed dispersal agents, and controllers of prey populations [1]. How the ecological processes that birds influence are linked across the landscape depends in part on birds’ habitat preferences, and the distribution of those habitats in the landscape [3-7].

Bird species richness is often associated with habitats altered by small mammal disturbances to the soil [8]. Some birds nest in cavities made by burrowing mammals (e.g. [9,10]). Higher bird species richness is observed within prairie dog (*Cynomys ludovicianus*) colonies in summer, compared to surrounding areas without these colonies [11,12]. Higher avian richness and abundance is also observed in grasslands with plateau pika (*Ochotona curzoniae*) burrows, compared to grasslands where they were eradicated [9,13]. Birds and small mammals often play different ecological roles. An association between birds and small mammals in habitats altered by small mammal activity could result in a coupling between different ecosystem processes at a landscape scale [2,8,14,15]. Granivorous birds tend to show different foraging site preferences and efficiencies, and different seed preferences and efficiencies, compared to small mammals [16-20]. Birds and mammals may also be complementary in that birds frequently act as seed dispersers [4,21,22], and small mammal disturbances to the soil can create bare or sheltered soil patches which enhance seedling establishment and recruitment [23,24]. Despite the many potential ecological interactions between birds and small mammals, we are not aware of studies that link small mammal ecosystem engineering effects with quantified avian ecosystem functions. Here we investigate how a small mammal’s disturbances affect the habitat preferences of birds in a semi-arid habitat, and the associated ecosystem-level effects.

**Figure 1:**
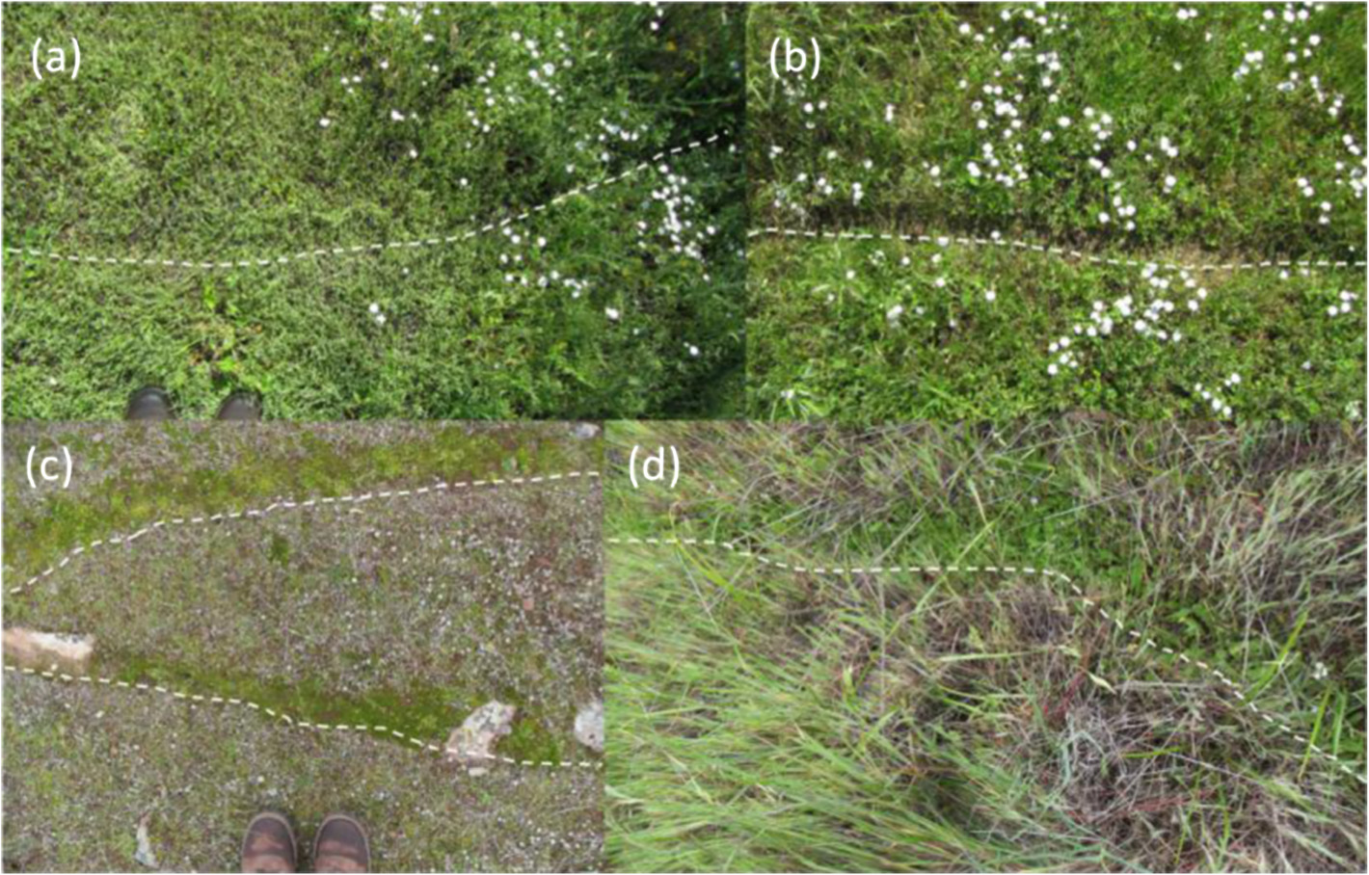
**Four images of degu runways.** Runways are marked with a dashed white line on the lower edge. **(a)** New runway, with plants trampled or pushed aside. **(b)** Established runway with bare earth in runway. **(c)** Winter, moss growing along established runways. **(d)** Early spring in a grassland, herbs growing along a runway which was likely formed the previous year. Photos **(c)** MR-B.

The degu, *Octodon degus*, is a small semi-fossorial social mammal native to the Mediterranean habitat of central Chile. Degus create colonies characterized by clusters of burrows with multiple entrances, all of which are connected by well-marked surface runways [25-27] (Figure 1). Sites with higher densities of runways are associated with higher herbaceous richness and diversity, forming a “lawn” of herbaceous plants in degu colonies due to herbivory next to runways and other ecosystem engineering effects [28]. Sites with high densities of degu runways also have higher bird feces counts (unpublished data, MR-B). Additional observations suggested that more bird species forage on degu lawns than on adjacent grasslands (pers. obs. M.R.-B.). Degu colonies alter the habitat at small scales, near to runways, whereas many birds have potentially landscape-scale ranges. We thus chose to examine the small scale sites where birds choose to forage within a landscape where a degu colony is present.

We investigate whether birds prefer to forage on and near degu runways, comparing their foraging effort across sites with a range of runway densities, including sites without runways. We measured granivory by granivorous and omnivorous birds. Because direct observation and quantification of naturally occurring bird foraging is excessively challenging and time consuming, we measure foraging effort by following the approach used in studies of granivory and giving up densities, of measuring amount eaten at feeding stations representing novel food sources in target habitat types [16,19,20]. We were able to exclude or discount non-avian granivores (see Methods). We were not able to directly measure foraging effort of insectivorous birds using the same technique, due to the high abundances of several non-avian diurnal and noctural carnivorous or insectivorous species at our study site (e.g. sigmodontid rodents, foxes, lizards, snakes), which we could not exclude or discount. As an alternative, since the majority of insectivores by abundance are omnivores at our site, we measure where foraging might be most diversified in terms of prey niche and nutritional content, by sampling invertebrate prey species richness across the same sites. Available evidence shows that omnivores direct foraging site selection to maximize access to immobile plants, opportunistically switching to mobile prey when encountered [29,30], see also [31]. Patches with complementary invertebrate and grain food sources provide more-efficient foraging opportunites to omnivores, and other things being equal, should be prefered [32-34]. Since we measure microhabitat/ substrate variables, and the larger ominivore present (the culpeo fox, *Lycalopex culpeaus*) is not observed to eat the bait used in this study, we account for or can ignore factors known to lead to prefences against these optimal foraging sites by omnivores [31,34]. For omnivores, this suggests that patterns of granivory over the landscape should correspond well to where insectivory takes place.

Invertebrate species richness and invertebrate abundances are expected to show opposite responses to avian predation on degu colonies. Invertebrates show higher richness near the disturbances made by many small mammals [35-37]. This can be a result of disturbance-related non-trophic interactions [38] or trophic interactions in which predation on a dominant competitor or intermediate predator allows more prey species to coexist [39]. Although improved habitat due to disturbance effects may lead to increased species abundances [38], the threat of predation by omnivores also attracted to the habitat can lead prey species to avoid these improved habitats [39]. In addition, widely foraging omnivores (such as birds) are expected to contribute to suppression of a common herbivore prey, such as herbivorous invertebrates [29]. Thus direct trophic interactions by birds are expected to reduce invertebrate abundances, through predation reducing population sizes, creating population sink areas, or forming a landscape of fear [40-42]. Overall then, we expect combined trophic and non-trophic effects on or near degu runways to increase species richness while decreasing species abundances. Classical optimal foraging theory predicts that foragers should optimize their site visitation rate to compensate for (and avoid causing) differences in prey abundance. Once modified to take into account the stoichiometric constraints and differences in prey handling costs relevant to omnivores, optimal foraging models become much more complex and may show mutliple equilibria [43-45]. Fitting such a model to our system is beyond the scope of this paper, and so we have not explicitly tested an optimal foraging hypothesis here.

In order to understand whether runway structure affects avian foraging, within each plot we compared foraging effort at an on-runway micro-site with an off-runway micro-site. We predicted that birds should show greater foraging effort, and invertebrate prey should be more diverse but less abundant, in plots with high runway density. We also expected to see greater foraging effort and more diversity but less abundance of invertebrates on runways, compared to off runways. Finally, we sought to relate some likely outcomes of common bird ecological functions to their foraging effort at the plot scale. The ecological functions and outcomes we examined included soil fertilization resulting from excrement deposition, shrub seed dispersal, and pest invertebrate population control, which correspond to supporting ecosystem services [1,22,46-48].

To our knowledge, this study represents the first time that avian activity and its outcomes on small mammal disturbance sites is quantified. This represents an advance in the resolution with which we can understand avian- small mammal non-trophic interactions, compared to simple measures of avian diversity. We show that avian foraging for grains and invertebrates was higher in plots where degu runways have higher density, and that this correlates with expected avian ecosystem service outcomes at the plot scale.

## Methods

### Research site

The study took place at the Estación Experimental Rinconada de Maipú (33°23′ S, 70°31′ W, altitude 495 m), a field station of Universidad de Chile, Santiago, central Chile. Our study site encompassed espinal (*Acacia caven* savanna) subject to anthropogenic fire and grazing by cattle and sheep, open grasslands, and denser matorral (evergreen shrubland), dominated by sclerophyllous shrubs and perennial herbs. Eighteen plots of 10 m x 10 m marked with flags were set up along the southeast, south-west and south facing slopes across thirteen small valleys in the folds of an inland extension of the coastal range (altitude 495 m), with variable degu runway densities. Most small valleys had at least one dry ravine dividing them down the middle, and each plot was separated from other plots by the ridge of a hill on one side and by a ravine or hill ridge on the other side. Two additional plots were placed in the lowland surrounding the hills, one in the east and one in the west, separated by a north–south dirt access road, for a total of 20 plots. The research site includes an area where another research group baits degu traps with oats, which attracts a large number of birds. Baiting continues six days a week throughout the winter and spring. None of our plots were within the area where baiting was taking place, and all plots were at least 100 m from the nearest baited area.

Each plot was surveyed to collect habitat data on 4 September 2011. In each plot we counted the abundance (number) of degu burrow entrances, abundance of degu runways, mean degu runway spacing, abundance of mounds made by the fossorial rodent the cururo (*Spalacopus cyanus*), tree abundance, shrub abundance, and percent estimated woody cover of each plot from shrubs and trees at 1 and 2 m from the ground. These data were used to categorize habitat type and characteristics. We expected these variables to influence where birds forage (e.g. degu burrows and runways, cururo mounds), or where they take cover and perch (woody plants). Degu runways can be counted individually as each is a distinct linear feature with clear beginning and end points, almost always connecting degu burrow entrances. Degu runway spacing in each plot was estimated by tossing a plastic frame in a semi-random (non-directed) manner ten times, each time measuring the distance to the nearest runway. We calculated the mean runway distance from these semi-random points for each plot, such that the resulting measure of mean runway spacing is an inverse measure of runway density or clustering. We had previously (2010) collected data on the slope and slope aspect of each site.

### Bird surveys

We surveyed bird species abundance and richness along twelve 100 m transects set up between the plots. We used the line transect technique, and used binoculars with a laser range finder (Bushnell Yardage Pro) to measure distances from the transect to sighted birds [49,50]. We did not record birds flying overhead. Each transect was characterized by habitat type, and the number of degu runways crossing the transect line was counted for each transect. Each transect was sampled twice, once in early September 2011 (late winter) and once in late October 2011 (mid spring), between dawn and 11:00, by one observer (M.R.-B.). For analysis, we used the DISTANCE program to calculate the abundance of birds [50] using habitat type and number of degu runways crossing the transect as strata [51]. We also used ANOVA to assess a linear model relating species richness to habitat type and number of crossing degu runways.

### Invertebrate prey richness and abundance

Invertebrates were collected in pitfall traps left open for 12 days between 16–28 October. Pitfall traps consisted of two 200 ml disposable plastic coffee cups, one placed inside the other, half covered with a flap of card [52]. The traps were placed in small pits, with the lip at ground level, and left open for one week to control for digging-in effects, as recommended [53,54]. Any animals (invertebrates or lizards) in the traps were released, and the traps were then filled with a 50:50 ethanol:glycerine mixture [55,56]. Two pitfall traps were placed in each plot, one at the edge of a degu runway (on-runway) and one at least 1 m from any runway (off-runway). Where a runway could not be found in the plot (in two plots), a runway within 2 m of the plot was used. In three plots we recovered a sample from only one pitfall trap. We excluded these three samples from our analyses of invertebrate abundances, but not from our analyses of species richness.

Invertebrates were collected, washed in water and alcohol, and stored in small plastic jars in 60:30 ethanol:water for identification. Identification was based on [57-59].

We sought to explain invertebrate taxon richness and total abundances using ANOVAs. Taxon richness (i.e. number of taxa at the order or family level) and total abundance (i.e. number of individuals summed across taxa) of each plot were considered independent measures. Our independent variables were abundance (number) of degu burrow entrances, abundance of degu runways, mean degu runway spacing, abundance of cururo mounds, tree abundance, shrub abundance, and percent woody cover of each plot from shrubs and trees at 1 and 2 m from the ground. We expected these variables to provide invertebrates with shelter (burrows, shrubs, trees), food (shrubs, trees), or general microhabitat heterogeneity with consequent access to different resources including high plant diversities [28] (runways, shrubs, trees, burrows). In order to understand how insectivore foraging patterns might relate to observed granivore foraging patterns, we also compared invertebrate abundances and species richness to our two measures of granivory effort in each plot (see below), using correlations.

### Granivorous foraging effort

To measure foraging effort by granivorous birds, we placed small metal dishes 11 cm in diameter in each plot, one on the edge of a degu runway (on-runway), and one at least one meter from any runway (off-runway). When it was not possible to find a runway within the plot, the on-runway dish was placed on a runway adjacent to the plot. Dishes were filled with approximately 25 g of rolled oats mixed with chili pepper to repel degus and other mammals, which unlike birds are sensitive to capsaisin [60] (1 kg oats: 200 g ground chili pepper). During five hours of preliminary observations we did not observe degus foraging on the spicy oats. Dishes were checked, weighed with an electronic weight (Acculab GS-200), and refilled if necessary each morning, at intervals of 24 hours, during four days between 20–24 August 2011. The amount of oats eaten was calculated as the difference between the weight of oats with which the dish was filled the previous day, and the current weight. In some cases the dishes of oats gained up to 2 g of water from dew. The net weight of moisture gained varied substantially between micro-sites and we were not able to find an effective way to set up a control for each dish that could be protected from consumption without affecting dew formation and evaporation rates. The recorded amount eaten may thus sometimes be an underestimate of ≤ 2 g. Because the amount recorded is thus a conservative estimate of amount eaten, it should reduce rather than inflate the likelihood of detecting the effects predicted for this measure. Missing oats due to occasional spillages were not treated as eaten.

In addition to measuring the amount eaten each day, we measured the number of days until each dish was first eaten from. We expect birds to forage more frequently at sites with greater prey abundances and/or greater ease of searching for prey [61]. Since birds are likely to continue to return regularly to check an area once a regular new food source is discovered there [62], long data sets on amount eaten at baited sites are not likely to reflect natural frequencies of site visitation. We assume that, since all sites were treated the same, dishes that are found and fed from first are in sites that are visited more frequently, on average, to search for food. We therefore continued the experiment only until a day passed when no new sites showed evidence of feeding. Some dishes were never eaten from, and for statistical analyses were treated as having been eaten from on the day after the experiment ended (e.g. five days until first forage). This gives a conservative measure of how often such sites are visited to forage.

Amount eaten and days to first forage for on-runway and off-runway dishes were compared with paired t-tests. We used linear models evaluated with ANOVA to determine the relationship beween days to first forage, degu runway density, and other habitat variables charaterizing the plots (see above), and also to determine the relationship between amount eaten and degu runway density and other habitat variables. Best models were selected based on the number of significant variables and the overall r value (the effect size) for the model.

### Avian functional traits

For the three identified ecosystem functions potentially carried out by birds at our study site (woody species seed dispersal, N and P deposition from feces, and invertebrate consumption), we identified a measure of ecosystem functioning outcome. Since the burrows and runways in degu colonies remain stable at our site over multiple years [63], we assume that ecosystem functioning outcomes will accumulate on degu colonies and that we can use measures of ecosystem functioning outcomes for avian behavior that would have accumulated over one or more years starting in the past. We predict that higher avian seed dispersal in plots should result in more small (young) shrubs, or a lower average percent cover per shrub at 1 m. We predict that where avian feces deposition increases in space, P and NO_3_^-^ concentrations in soil should also increase in each plot [47]. Soil samples were taken in 2010 and the methods of sampling are described in [28] and Root-Bernstein et al. (submitted). It is unlikely that soil [P] and [NO_3_^-^] vary significantly between two sequential years [64]. Finally, to measure invertebrate consumption outcome, we classified our invertebrate data set into potential agricultural pest (phytophagous) and non-pest taxa, and calculated the percent by abundance (i.e. percent of total number of individuals) of pests in each site. We predict that as avian pest invertebrate consumption increases in each plot, percent pest abundance will decrease.

To quantify these three ecosystem functions (woody species seed dispersal, N and P deposition from feces, and phytophage insect population control), we developed an index of the functional trait impact (FTI) at the plot scale. The FTI approximates how often birds actually visit each plot, carrying out ecosytem functions (see Additional file 1). To determine which bird species at our site had which functional traits (Additional file 1), we consulted standard literature on these species traits and habits [65]. We used correlations to relate the FTI at each plot to the measures of ecosystem function outcome. Since our predictions are directional we report one-tailed p values, accepting p < 0.05. We anticipate only a weak correlation due to lack of precise data on the activities of each species in each plot over the past year, and also due to the small spatial scale relative to the processes studied. This part of the research is thus exploratory.

For all statistical tests except the tests in DISTANCE, statistics were run in R, and p values were calculated with Graphpad Software (http://www.graphpad.com). For all tests except the analysis in DISTANCE, we used an ANOVA approach, prefering models with the largest effect sizes (overall model r) and the largest number of significant variables. DISTANCE reports only AIC values.

## Results

### Avian abundance and richness

In total there were 202 observations of birds along the transects, with an effort of 370 minutes. The best fit model of abundance in DISTANCE assumed a common detection function across habitat type and number of degu runways (AIC: 1793.4). The calculated density of birds was 0.009 birds per m^2^ (nearly one bird per hectare), with a detection radius of 44.72 m and a mean cluster size of 1.69 birds. Of 19 bird species observed, one (5%) is a carnivore, seven (37%) are insectivores, six (32%) are granivores, and five (26%) are omnivores. Average avian richness on transects across September and October samples was not explained by the number of degu runways crossing the transect, nor by habitat type (Table 1). By abundance, 8% of the observed avian population (averaged across samples) were insectivores, 25% were omnivores, 57% were granivores and 10% were carnivores.

**Table 1:** ANOVA of average avian richness on transects

**Variables**	**df**	**Sum sq.**	**F**	**p**
Number of degu runways	1	7.9	4.36	0.075
Habitat type	3	6.9	1.26	0.36
Residuals	7	12.7		

Overall model r = 0.66.

### Invertebrate prey abundance and richness

In total we trapped 1040 individual invertebrates representing 54 taxa from 15 orders (Table 2). Across plots, invertebrate abundance increased with invertebrate taxon richness (r = 0.798, df = 16, p < 0.0001). The best model for taxon richness across plots included the interaction between mean degu runway spacing and woody cover at 2 m from the ground, and the number of shrubs (overall r = 0.63, Table 3). The best model for total invertebrate abundance across plots included the variables mean degu runway spacing, valley, and the interaction between number of runways and slope aspect, with more runways and lower invertebrate abundances on SE facing slopes and vice versa on SW facing slopes (overall r = 0.99, Table 3).

**Table 2:** Invertebrate taxa observed and characteristics

**Taxa**	**Total abundance**	**Saprophyte or other decomposer**	**Pollinator**	**Predator**	**Phytophage**
Coleoptera: Melyridae	156		+	+ (larva)	
Coleoptera: Bostrichidae	101	+			
Coleoptera: Anobidae	1	+			
Coleoptera: Ptinidae	1	+			
Coleoptera: Tenebrionidae	35	+			
Coleoptera: Tenebrionidae (larva)	7	+ (larva)			+
Coleoptera: Elateridae	1		+		+ (larva)
Coleoptera: Carabidae	19			+	
Coleoptera: Glaphyridae	22		+		+ (larva)
Coleoptera: Staphylinidae	4	+		+	
Coleoptera: Lathridiidae	2	+	+	+	
Coleoptera: Coccinellidae (larva)	15			+	
Coleoptera: Coccinellidae	3			+	
Coleoptera: Chrysomelidae	2				+
Coleoptera: Curculionidae	3				+
Coleoptera: Mordellidae	2	+ (larva)	+		+ (larva)
Hymenoptera: Sphecidae	2		+	+	
Hymenoptera: Pompilidae	1		+	+	
Hymenoptera: Calcidoidea, Fam?	14		+	+	
Hymenoptera: Apoidea, Fam?	13		+		
Hymenoptera: Calcidae	0			+	
Hymenoptera: Mutillidae	2			+	
Hymenoptera: Chrysididae	2		+	+	
Hymenoptera: Fam?	0				
Hymenoptera: Formicidae	91			+	
Lepidoptera: Tortricidae	13		+		+ (larva)
Lepidoptera: Noctuidae (larva)	9		+		+ (larva)
Lepidoptera: Fam?	1		+		+
Diptera: Fam?	2				
Diptera: Ascilidae	2			+	
Diptera: Bombyliidae	0		+	+	
Diptera: Mycetophilidae	6	+			
Diptera: Phoridae	2		+	+	
Diptera: Tephritidae	1		+		+
Diptera: Calliphoridae	1	+	+		
Diptera: Sciaridae	12	+			
Diptera: Muscidae	1	+	+		
Diptera: Drosophilidae	0	+			
Diptera: Agromyzidae	1				+
Hemiptera: Lygaeidae	64			+	+
Hemiptera: Miridae	18			+	+
Hemiptera: Aphididae	6				+
Hemiptera: Fam?	2				
Hemiptera: Cicadellidae	65				+
Orthoptera: Fam?	6				+
Orthoptera: Tettigoniidae (nymph)	16				+
Orthoptera: Acridiidae	10				+
Orthoptera: Acridiidae (nymph)	2				+
Orthoptera: Gryllidae	1	+			
Psocoptera	1	+		+	
Thysanoptera	2				+
Thysanura	84	+			
Collembola	21	+			
Isopoda	5	+			+
Opilionida	22			+	
Arachnida: Araneae	31			+	
Arachnida: Solifugae	2			+	
Acaridida	131	+		+	
Pseudoescorpionida	1			+	

List of all invertebrate taxa observed, their total abundance across plots, and whether species in each taxonomical group present important functional traits. Fam? indicates that the taxa could not be identified at the family level.

**Table 3:** Invertebrate taxon richness and total abundances

	**Invertebrate taxon richness**					**Invertebrate total abundances**			
**Variables**	**df**	**Sum sq.**	**F**	**p**	**Variables**	**df**	**Sum sq.**	**F**	**p**
Mean degu runway spacing	1	51.9	2.17	0.16	**mean degu runway spacing**	**1**	**556**	**2845.08**	**0.012**
Woody cover at 2 m	1	3.6	0.15	0.70	**Runway abundance**	**1**	**107**	**548.42**	**0.03**
Shrub abundance	1	0.4	0.02	0.90	**Slope aspect**	**1**	**1862**	**9525.09**	**0.007**
**Runway spacing x**	**1**	**180.4**	**7.55**	**0.01**	**Valley**	**11**	**33274**	**15471.41**	**0.006**
**Woody cover**					**Runway spacing x aspect**	**1**	**436**	**2231.66**	**0.01**
Residuals	15	358.6			Residuals	1	0		
r = 0.63					r = 0.99				

ANOVAs for invertebrate taxon richness across plots (left) and invertebrate total abundances (number of individuals of all taxa) across plots (right). Significant variables are shown in bold.

### Avian foraging effort

In 17 of the 20 plots, on-runway and off-runway dishes were first foraged from on the same day. We therefore modeled only time to find the on-runway dish. The best model explaining the number of days until the first foraging was detected from the on-runway dish in each plot included the number of cururo mounds, mean degu runway spacing, the interaction between arboreal cover at 2 m and the slope, and a variable identifying the valleys in which plots are located (Table 4). Time to discover the on-runway dish decreased as cururo mounds, degu runway density, and arboreal cover increased, and as slope decreased. The valley variable indicates that some of the variance in time to discover the dishes was not explained by habitat variables but may be attributed to spatial autocorrelation among adjacent valleys affecting avian foraging patterns. The model was not improved by including the valley variable explicitly as an error term.

**Table 4:** ANOVA of the number of days to forage from the on-runway dish

**Variables**	**df**	**Sum sq.**	**F**	**p**
**Cururo mounds**	**1**	**6.2**	**3.9 e^31**	**2.2 e^-16**
**Degu runway density**	**1**	**6.5**	**4.07 e^31**	**2.2 e^-16**
**tree cover, 2 m**	**1**	**0.9**	**5.6 e^30**	**2.7 e^-16**
**Slope**	**1**	**4.8**	**3.0 e^31**	**2.2 e^-16**
**Valley**	**13**	**44.8**	**2.2 e^31**	**e.e e^-16**
Tree cover, 2 m x slope	1	0		0.51
Tesiduals	1	0		

Significant variables are in bold. Overall model r = 1.0.

The total amount eaten over the course of the experiment did not differ between on-runway and off-runway dishes (t = 1.45, df = 19, p = 0.16, paired t-test). We summed the amount eaten on and off runway for each plot and modeled the amount eaten as a function of habitat variables. The best model included mean degu runway spacing and an interaction between the number of shrubs and a variable identifying the valleys where plots are located (Table 5). The amount eaten in each plot increased with degu runway density and decreased with shrub cover. The variable “valley” suggests some spatial autocorrelation, since the valleys were numbered sequentially from west to east. Thus, an effect of valley is an effect of spatial contiguity in the landscape.

**Table 5:** ANOVA of the total amount eaten summing on-runway and off-runway dishes

**Variables**	**df**	**Sum sq.**	**F**	**p**
**Degu runway spacing**	**1**	**7695**	**51.74**	**0.006**
Shrub abundance	1	106	0.71	0.46
**Valley**	**13**	**48798**	**25.24**	**0.01**
Shrub abundance x valley	1	1278	8.59	0.06
Residuals	3	446		

Significant variables are in bold. Overall model r = 0.99.

To understand how insectivore foraging patterns might be related to granivore foraging, we compared the abundances and taxon richness of invertebrates at each site to the granivorous foraging effort at each site. By abundance, invertebrate-eating birds (insectivores plus omnivores) were 75% omnivores, suggesting that there should be significant overlap between locations for granivory and insectivory. Invertebrate abundance was not significantly lower in plots where more oats were eaten (r = -0.377, df = 16, p = 0.12). Invertebrate abundance in plots increased significantly with time to first forage from oat dishes (r = 0.613, df = 16, p = 0.0068). By contrast, invertebrate taxon richness was not related to either the amount of oats eaten or to the number of days to first forage at the plot (amount eaten: r = -0.064, df = 19, p = 0.78; days to first forage: r = 0.079, df = 19, p = 0.73).

### Avian functional traits

Three sets of functional traits recorded for the birds on the transects are shown in the Additional file 1. These functional traits correspond to the three common avian ecosystem functions that we measured (woody species seed dispersal, N and P deposition from feces, and pest invertebrate consumption). FTI indexes for each ecosystem function are also shown in the Additional file 1. The seed dispersal FTI index showed a moderate significant negative correlation to average shrub percent cover at 1 m, after a shrub-cover outlier was removed (Grubb’s test to remove an outlier, Z = 2.71, p < 0.05; r = -0.389, df = 18, p = 0.041). Thus, the average size of shrubs decreased with increased FTI. The feces deposition FTI index showed a moderate significant positive correlation to NO_3_^-^ concentration in the soil, after an [NO_3_^-^] outlier was removed (Grubb’s test to remove an outlier, Z = 2.71, p < 0.05; r = 0.380, df = 18, p = 0.049), but not to P concentration in the soil (r = 0.135, df = 19, p = 0.280). The insect control FTI index showed no correlation to the herbivorous insect percent abundance in plots (r = -0.175, df = 19, p = 0.224). However, we noticed that the plots where the FTI index was zero (N = 7) appeared to show a discontinuous pattern of distribution from plots where FTI was nonzero. Where the FTI was zero (i.e. birds do not visit), abundances were uniformly low. In all other plots, abundances were abruptly high for low foraging, and then declined with the foraging index. Looking only at plots were FTI was non-zero (i.e. plots where birds visit), we found that the insect control FTI was significantly negatively correlated to herbivorous insect percent abundance (r = -0.515, df = 12, p = 0.03).

## Discussion

Our results support the hypothesis that birds choose degu colony areas with high runway densities as preferred foraging sites within a heterogeneous landscape in central Chile. Birds found novel food sources faster, and ate more, at plots with higher densities of degu runways. They also preferred to forage on flatter plots with trees and cururo mounds, but tended to forage less on plots dominated by shrubs. Our interpretation is that plots that birds visited first are in habitat patches generally visited more often. This is because during a random time interval, a site that is visited more frequently on average has a higher probability of being visited more frequently during the interval, and earlier in the interval, than a site that is visited less frequently on average. After foraging at a plot was recorded, this activity was almost always recorded through all subsequent days. Thus birds appear to return to these preferred experimental patches. This implies that the longer a plot remained unvisited, the less frequently it yields foraging rewards under non-manipulated circumstances. The inclusion of the valley variable in the best ANOVA model suggests that birds forage at plots near to where they were already foraging, influenced by the topography of the research site. Seven plots (or 35%) where not used for foraging, and these plots were interspersed geographically with contiguous areas where foraging did take place. This is consistent with birds regularly visiting prefered locations, and skipping other locations, due to their habitat characteristics [9,11-13]. Birds failed to forage in some plots with relatively high degu runway density, showing that all the significant explanatory variables (degu runways, trees, cururo mounds, flat terrain and contiguity) contributed to avian foraging patterns across the landscape. Amount eaten in visited plots was controlled primarily by degu runway density and also by lack of shrub cover.

We observed that birds are not more abundant in specific habitat types or degu runway densities within the landscape. When observed on transects, birds were performing a range of activities in addition to foraging, such as singing, vigilance, or resting. This suggests that birds move to areas with higher densities of degu runways, and/or trees and cururo mounds to forage. Degu runways provide a network of small edges and linear clearings in the herbaceous substrate, which could affect foraging efficiency by making seeds and invertebrates more visible at runway edges and clearings. In addition, degu lawns in areas of high runway density typically consist of very short herbs (pers. obs. MR-B) probably due to herbivory [66]. This could improve foraging efficiency by providing reduced physical or visual obstruction, which simultaneously may aid both prey detection and predator detection [67-69]. However, there was no evidence that avian foraging activity was influenced by the small-scale structure of runways, as they did not forage more or earlier at dishes on runways compared to off runways. Thus, when foraging, birds were sensitive to habitat differences at the plot scale (100 m^2^) but not at the scale of degu runways (≤ 1 m^2^).

Avian foragers appear to be attracted to the disturbances of another small mammal present at the site, cururos. Cururo mounds are ephemeral, lasting less than a year, are about 400 cm^2^, and occur in clusters of between half a dozen to hundreds which may appear throughout spring and summer (pers. obs. MR-B, [70]). The role of these mounds in attracting foragers deserves further attention, as does their effect on ecological processes, which is likely to be different from that of degu runways due to their different shapes, sizes, permanence times, and effects on the soil [38]. Cururo mounds and degu runways may be linked ecologically, as new degu burrows often appear to be modified cururo tunnels (pers. obs. MR-B).

Although we were not able to directly measure insectivorous foraging effort, we were able to measure invertebrate prey taxon richness and total abundances in the same plots where we measured granivory. Like herbaceous plant richness, invertebrate prey richness increases with increasing degu runway density, on southwest facing slopes and with greater woody cover [28]. Herbivorous and nectarivorous invertebrates may be attracted to degu runways due to the increased herbaceous plant richness associated with runways [28,71]. This would explain the abundance of Melyridae, whose adults are polinophagous [72] and visit a wide range of herbaceous and woody species common in steppes of semiarid Chile [73]. Saprophytes and other detritivores (e.g. Tenebrionidae) could be attracted to feces or shrub litter that accumulates near burrows ([74], pers. obs. MR-B). The high abundance of Bostrichidae, whose larvae and adults are strict saproxylic consumers of dead wood in the early stages of decomposition [75] supports the association between degu runway densities, woody vegetation, and invertebrate species richness (see also below). In turn, invertebrate predators and parasitoids could be attracted to prey and favorable hunting habitat [71,72]. Generally, invertebrate niches should be affected by the microhabitat heterogeneity provided by the small-scale edge structures of runways [76-78].

Invertebrate taxon richness was strongly correlated with total invertebrate abundances across plots. We found that avian granivorous foraging effort, measured either as days to find the food dish, or amount of oats eaten, was not related to the invertebrate prey taxon richness. As expected however, plots that were visited sooner by birds during granivory trials showed significantly lower invertebrate prey abundances. Consistent with research on omnivorous foraging, we interpret this pattern as resulting from omnivorous birds foraging opportunistically for both grains and invertebrates in the same habitat patches, which in turn depress invertebrate abundance at the most frequently visited foraging sites [30,41]. However, the observed depression in invertebrate abundances must be due to non-avian as well as avian insectivory. This suggests either that birds are the main insectivores in this community, or that other insectivores have similar foraging site preferences. Among strictly insectivorous species, *Sturnella loyca* tend to forage off degu colonies in grassland habitats, while *Leptasthanura aegithaloides*, *Mimus tenca* and *Troglodytes musculus* forage in trees, which was a significant factor for granivorous foraging as well. *Vanellus chilensis* are often observed on degu lawns, and *Pteroptochos megapodium* and *Scelorchilus albicolus* may nest in degu burrows, all of which are then likely to forage on degu colonies. Although we could not obtain direct evidence of where avian insectivorous foraging effort is focused, our data indirectly suggest that degus influence insectivorous foraging as well as granivorous foraging, through non-trophic ecosystem engineering effects on the plant community [28] and consequent provision of favorable invertebrate habitat.

Previous studies have not linked habitat preferences of vertebrate consumers while performing their ecosystem functions to how those functions are distributed at such a fine scale (compare [7,80]). The ability to quantify plot-scale functional diversity at very small scales could be a useful tool to communicate to land managers and farmers how changes in habitat structure impact local benefits from ecosystem services [7,79,80]. We found that for all three ecosystem functions measured, we could detect a possible patch-scale signature of ecosystem function. Smaller shrubs were more frequent where seed eating birds forage more, which could be a result of previous shrub seed dispersal by birds. NO_3_^-^ concentration increased where ground-foraging birds forage more, which could be the result of defecation onto the ground. As insectivore foraging increased, phytophagous invertebrates became proportionately less abundant. While a causal inference is supported by many studies showing that birds have such effects [1], we cannot rule out that these correlations could be the result of habitat selection by birds for small shrubs, NO_3_^-^ rich soils, and low phytophagous invertebrate abundances, rather than feedback from avian ecosystem functions. The FTI also makes several assumptions that may reduce its accuracy. One is the assumption that foraging activity, as measured by the oat-baited dishes, represents foraging in equal proportion by all birds present in the landscape. A second simplifying assumption is that birds visit the plots only during foraging activity. Futher development of small-scale measures of functional diversity outcomes could yield data with higher resolution.

## Conclusions

We quantified the foraging activities of both invertebrate-eating and granivorous birds, finding that they forage more in plots where degu runways have higher density, in flat areas with trees and cururo mounds. This foraging activity on plots with degu runways was in turn correlated to expected outcomes of avian ecosystem services, representing an advance in our ability to measure ecosystem service outcomes at small spatial scales. We predict that this approach can be developed to contribute to detecting other small-scale signatures of ecosystem functioning, and to understanding the impact of ecosystem engineers on key long-distance linkage species, whose movements across the landscape contribute to mobile-agent-based ecosytem service (MABES) dynamics over larger areas [2,7].

### Availability of supporting data

The data sets supporting the results of this article are available in the Google Drive repository, https://drive.google.com/file/d/0B3ASin6EE6tlSE9CcEdqbElrUDg/edit?usp=sharing

## Competing interests

The author(s) declare that they have no competing interests.

## Author’s contributions

MR-B designed the experiment, carried out the fieldwork, and drafted the manuscript. AF identified the invertebrates and added relevant material to the manuscript. JA and LAE helped draft the manuscript. All authors read and approved the final manuscript.

## Authors’ information

MR-B is a post doctoral fellow researching ecosystem services in central Chile. AF is completing a PhD on invertebrate responses to fragmentation. JA is an adjunct professor with many years experience in studying avian and plant ecology in Chile. LAE is an associate professor researching degu sociality.

## Supplementary Material

Additional file 1 Calculation of the functional trait impact per plot. Click here for file
