# Supplementary material for: Avian ecosystem functions are influenced by small mammal ecosystem engineering
Source: BMC Res Notes. 2013 Dec 20;6:549. doi: 10.1186/1756-0500-6-549 (PMC3878134; doi:10.1186/1756-0500-6-549)
Supplement: Additional file 1 — Calculation of the functional trait impact per plot. [file 1756-0500-6-549-S1.docx]

Supplementary Information

In order to assess how often birds actually visit each plot, carrying out in the plot the ecosytem functions attributed to them via their traits, we use our data from the granivory trials. The formula for this index draws on our available data and is thus is study-specific. It creates a strictly relative measure of how much birds with each functional trait actually visit each plot, carrying out the ecosytem functions attributed to them via their traits. The functional trait impact (FTI) index is defined as FTI = Σ (A * T) * V,

where A is the mean species abundance (averaged across early and late spring) of each species, summing all transects; T is the trait value (1 = has the trait, 0 = does not have the trait) for each species for the functional trait in question (Table 1); and V is the visitation pressure, defined as:

V = F / D,

where F is the mean amount consumed during the avian granivorous foraging effort trials per day, excluding days before the first food dish at the plot was first found; and D is the number of days until the first food dish at the plot was first foraged from. The FTI index thus measures the functional traits, weighted by abundances, in the wider landscape (across all transects), scaled to the visitation pressure (during foraging) by birds at each plot (see Figure 1).


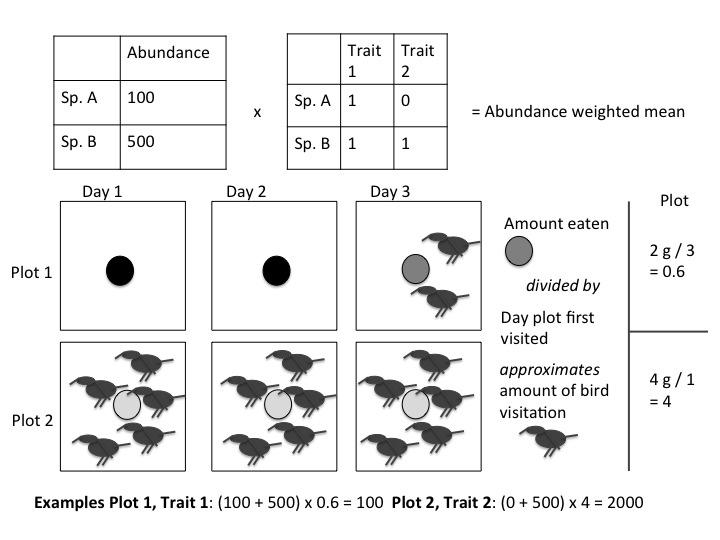


Figure 1. An example of how the FTI is calculated, showing what it aims to approximate and how.

Table 1. Functional trait values and associated FTI indices.

|  | **Functional traits** | | |  | |
| --- | --- | --- | --- | --- | --- |
| **Species** | eat shrub seeds/fruits | eat insects | ground forager | |  |
| *Colaptes pitius* |  | 1 | 1 | |  |
| *Columbina picui* | 1 | 1 | 1 | |  |
| *Diuca diuca* | 1 |  | 1 | |  |
| *Geositta cunicularia* |  | 1 | 1 | |  |
| *Leptashenura aegithaloides* |  | 1 |  | |  |
| *Mimus tenca* | 1 | 1 |  | |  |
| *Molothrus bonariensis* | 1 | 1 | 1 | |  |
| *Parabuteo unicinctus* |  |  |  | |  |
| *Phrygilus alaudinus* | 1 |  | 1 | |  |
| *Phrygilus fruticeti* | 1 |  | 1 | |  |
| *Phrygilus sp.* | 1 |  | 1 | |  |
| *Phrygilus unicolor* | 1 |  | 1 | |  |
| *Pteroptochos megapoides* |  | 1 | 1 | |  |
| *Scelorchilus albicollis* |  | 1 | 1 | |  |
| *Sturnella loyca* | 1 | 1 | 1 | |  |
| *Troglodytes musculus* |  | 1 |  | |  |
| *Turdus falklandii* | 1 | 1 | 1 | |  |
| *Vanellus chilensis* |  | 1 | 1 | |  |
| *Zonotrichia capensis* | 1 |  | 1 | |  |
|  | **Index of FTI** | | | |  |
| **plot** | shrub seed dispersal | pest insect consumption | feces deposition | |  |
| 1 | 185 | 224.7 | 558.5 | |  |
| 2 | 0 | 0 | 0 | |  |
| 3 | 0 | 0 | 0 | |  |
| 4 | 2018.75 | 1631.1 | 4053.6 | |  |
| 5 | 418.75 | 338.3 | 840.8 | |  |
| 6 | 0 | 0 | 0 | |  |
| 7 | 0 | 0 | 0 | |  |
| 8 | 0 | 0 | 0 | |  |
| 9 | 0 | 0 | 0 | |  |
| 10 | 3150 | 1272.6 | 3162.6 | |  |
| 11 | 3400 | 1373.6 | 3413.6 | |  |
| 12 | 4537.5 | 1833.1 | 4555.6 | |  |
| 13 | 5387.5 | 2176.5 | 5409 | |  |
| 14 | 1675 | 676.7 | 1681.7 | |  |
| 15 | 500 | 202 | 502 | |  |
| 16 | 400 | 479.7 | 1192.2 | |  |
| 17 | 0 | 0 | 0 | |  |
| 18 | 1600 | 646.4 | 1606.4 | |  |
| 19 | 2350 | 1893.7 | 4706.2 | |  |
| 20 | 1450 | 585.8 | 1455.8 | |  |

Above, the functional traits possessed by each of the species observed in avian transects in our study site. Below, the values for each plot of the index of plot-scale functional diversity for three ecosystem functions. The functional trait above in each column corresponds to the ecosystem function in the column below.
